# Integration of a peer practitioner in a hospital unit for patients with psychotic disorders: an exploratory qualitative study

**DOI:** 10.3389/fpsyt.2023.1244433

**Published:** 2023-09-26

**Authors:** Pierre Lequin, Caroline Suter, Roxane Mazallon, Rachèle Brodard, Lilith Abrahamyan Empson, Bruno Robalo, Philippe Conus, Alexandra Nguyen, Jérôme Favrod

**Affiliations:** ^1^School of Nursing La Source, University of Applied Sciences and Arts of Western Switzerland (HES-SO), Delémont, Switzerland; ^2^Department of Psychiatry, Service of General Psychiatry, Lausanne University Hospital, University of Lausanne, Lausanne, Switzerland

**Keywords:** peer practitioner, psychiatric hospital, workplace integration, collaboration, qualitative study, recovery

## Abstract

**Introduction:**

Studies on the integration of peer mental health practitioners (PMHP) in hospitals are sparse, despite significant benefits being reported for patients and professionals. The integration of PMHP requires the consideration of several parameters and a change in the culture of care. This study aims to understand the impact of the integration of a PMHP in a hospital unit caring for patients with psychiatric disorders.

**Methods:**

A qualitative content analysis of three focus groups with the interdisciplinarity team were conducted. A consulting PMHP was integrated into the entire research process.

**Results:**

Data analysis revealed five main themes: the importance of integration, benefits for patients linked to the identification process, benefits for the team and institution, potentials risks, and perspectives.

**Discussion:**

The study was conducted in a hospital setting with patients suffering from severe psychiatric disorders associated with behavioral disturbances. The benefits reported in the results outline the feasibility of PMHP integration in an acute psychiatric care setting. Nevertheless, further formalization of the PMHP role is required to minimize possible areas of tension between respective fields of activity of each professional.

## 1. Introduction

Peer mental health practitioners (PMHP) are people who have experienced mental illness and have been trained to support the recovery of others from an experiential standpoint. They participate in individual and group care, research, and training. A 2012 Cochrane review of 11 randomized controlled trials (RCTs) concluded that care teams with PMPH resulted in identical clinical outcomes compared with those without (1). The review reported a low level of evidence on the decreased use of emergency services but no difference in the rate of re-hospitalization. Another meta-analysis confirmed these results, but noted that the number of eligible studies remained low because of the heterogeneity of the variables studied (2). However, positive effects on the feeling of hope, recovery, and empowerment, as well as an improvement in quality of life, have been reported (3). Interventions by peer practitioners appear to be superior to those conducted by non-peer professionals in terms of these four variables (4). These results are supported by a meta-synthesis of qualitative data, revealing that peer practitioners are a source of hope because they are perceived as role models by patients (5). In addition, they help to engage and motivate patients to continue their treatment, and their interventions allow patients to develop their social network. Finally, it reports that patients find it easier to make contact with peer practitioners because they have less professional distance (5). This allows them to act as a facilitator of the relationship between patients and professionals (6). Professionals who have worked with peer practitioners have stated that this has allowed them to develop a better understanding and more empathy towards patients and form a more positive view of recovery (5, 6).

Most of the previous studies have been conducted in the community setting, although definite benefits have been reported for patients in those conducted in hospitals: improved sense of hope contributing to the perception that recovery is possible; reduced stress, anxiety, and feelings of loneliness; support with activities of daily living; and person-centered rather than illness-centered interaction (7–9). In addition, a decrease in the use of restraint, both in terms of frequency and duration, has also been observed (9–12). With regard to these measures, PMHP were associated with preventive interventions by including the patient in their treatment plan, by a systematic review of the measure during the intervention, and by a debriefing with the patient after the measure (10–13). Hospital professionals are also benefitted by the presence of a peer practitioner in helping to improve patient compliance with the drug treatment, particularly during its introduction. The peer practitioner thus supports the health care team, with team meetings facilitating the adoption of a more respectful language towards patients (6).

The inclusion of peer practitioners in health care teams requires the consideration of several parameters at the institutional level and in relation to the team members (14). First, regarding the culture of care, it is recommended that the team be familiar with the values of recovery and that it perceives the experiential experience of illness as a resource that can support the recovery of patients (14). In addition, the team should be prepared for the arrival of a peer practitioner by receiving information on their roles and identifying their potential scope of intervention. Furthermore, a description of the role of the peer practitioner in the team is essential, while avoiding its over-formalization (15, 16). Indeed, the terms of reference must be co-constructed and adjusted in partnership with the peer practitioner, considering their skills and perspectives (15). The terms of engagement, remuneration, and supervision must be clarified at the institutional level (14). Finally, it is important to be attentive to the stress generated by the pressure of success on the peer practitioner when they join the team (16).

Considering the benefits described above, we employed a PMHP at our hospital. The current study thus aimed to understand the impact of the integration of peer practitioners in a psychiatric hospital care unit as perceived by the multidisciplinary team. By conducting a study on the integration of a PMHP in a hospital unit, healthcare organizations can gain a deeper understanding of the challenges involved. This knowledge can inform the development of appropriate policies, protocols, and support mechanisms to ensure the successful deployment of experiential experts in the hospital environment, ultimately leading to improved mental health care for patients.

## 2. Materials and methods

### 2.1. Design

An exploratory qualitative design was recommended, given the limited amount of research conducted in hospitals with patients with psychotic disorders. The balance between academic researchers and people with lived experience in the design and implementation of this study was constructed as follows: CS was included from the initial stage of study design and her perspective was taken into account all along the course of the study. Decisions were taken collectively, considering the different expertise and points of view. She has been part of a research team for 5 years. Both PMHP (CS and RM) were properly paid for their work. Their contribution is acknowledged in publications and academic presentations.

### 2.2. Setting

The study was conducted in an acute psychiatric unit of a Swiss university hospital. This unit admits patients suffering from psychotic disorders, who present an acute symptomatology often associated with behavioral disturbances. The average length of stay is 20 days, and the unit can provide seclusion care.

### 2.3. Ethical considerations

This study is outside the scope of the Swiss Human Research Act because no personal data concerning human diseases and the structure and function of the human body were collected. Therefore, this study did not need to be authorized by Swiss ethics. All participants provided their written informed consent. Focus group recordings were transcribed and anonymized verbatims were entered in NVivo 1.7.1.

### 2.4. Sample and data collection

The professionals of the multidisciplinary team (*n* = 13) of the unit with a full- or part-time position were invited to participate in the study. They gave their consent after they were provided with oral and written information about the study. Apart from one person, all agreed to participate in the study and gave their written consent (*n* = 12). Prior to the arrival of the peer practitioner, they were invited to participate in preparatory work sessions to welcome the peer practitioner and discuss the issues surrounding their work in the team. The PMHP began working in the unit on March 1, 2021. Three focus groups were conducted 6 months after their arrival in September 2021. Prior to the focus groups, a questionnaire constructed by the research team was sent to all the participating professionals to conduct an initial assessment of their satisfaction. Open-ended questions allowed them to express the initial benefits perceived and the difficulties encountered. These elements were used to enrich the discussion during the focus groups.

The focus group method was selected to facilitate the expression of the participants’ perspective to limit the impact on the organization of hospital work and to save time. Focus groups were prepared and conducted following Doody et al. (17). The focus groups and interviews were conducted by PL and CS because of their complementary skills and expertise. The head nurse of the unit, the senior physician, and the nurse referent were all integrated into the same focus group to avoid the risk of inhibiting exchanges due to their hierarchical function. The referring nurse provided close support to the integration of the PMHP. A common tailor-made interview guide, consisting of different sections, was constructed based on the literature review and the responses to the above-mentioned questionnaire. The questionnaire begins with an open-ended question that seeks to assess the experience of staff members in integrating the peer practitioner function in the hospital. The perceived benefits for patients, non-peer professionals, and the institution were then investigated, along with the challenges and difficulties encountered. Finally, the participants were asked about their vision of recovery. Because of their hierarchical position, the head nurse and the senior physician were asked about team dynamics. For each question, based on the literature and according to the answers to the questionnaire, follow-up questions were predefined.

### 2.5. Data analysis

The three focus groups were recorded and transcribed. The data were then anonymized and analyzed using NVIVO (QSR International; Burlington, Massachusetts, United States) software by employing the content analysis method following Braun and Clarke’s approach (18). The material was first read and reread by the principal investigator and consulting peer practitioner in its entirety to take ownership of the content and identify its main ideas. The data were then divided into units of meaning and coded for each section of the questionnaire. The grouping of codes was subject to inter-coder agreement between the researcher and researcher peer practitioner to identify the themes. A third researcher (JF) was then brought in for confrontation and discussion until consensus was reached. The main themes identified included benefits for patients and the health care team, risks and concerns, integration, context of care, perspectives, and the skills of the peer practitioner.

## 3. Results

A total of 12 stakeholders were recruited, including an associate physician (*n* = 1), a physician (*n* = 1), a head nurse (*n* = 1), unit nurses (*n* = 7), a social worker (*n* = 1), and a health care and community health assistant (*n* = 1). The results were organized into five categories: integration of the function, benefits for the patients, benefits for the team and the institution, risks, and development perspectives.

### 3.1. Integration

The working sessions prior to the engagement of the peer practitioner were viewed as important by the caregivers in the unit to understand the role and its scope of practice. The involvement of the consulting peer practitioner was praised:

*It was important to understand the function, the specifications in quotes. For me it was important, yes.* (R1).

*And then the fact that there was the consultant peer practitioner, for me too that was an added value to the preparation and integration afterwards.* (R7).

These sessions allowed caregivers to express their questions and fears related to professional secrecy, addressed their concerns about the method of communication, and clarified restraint care arrangements.

*I remember that there was a whole series of questions about confidentiality of function, medical confidentiality, how this person could welcome the way caregivers could talk about patients outside the presence of the patient. What would be his feelings, his experience, or his view of our profession and particularly regarding restraint measures?* (R12).

This was followed by the smooth integration of PMHP into the care unit. The stability of the staff and a caring work environment were seen as facilitating factors:

*I thought it went well; quite easily actually. I do not know if it’s related to the preparation, but I have the impression of a great naturalness in the arrival of the peer practitioner. We very quickly forgot that she was new, that her function was new. I think she became part of the team very quickly.* (R9).

*It’s true that we are a pretty stable unit. I think that plays a lot into the integration of a peer practitioner.* (R4).

Similarly, the peer practitioner’s interpersonal skills and enterprising spirit were also seen as beneficial to integration:

*She is someone who is very curious, who asked a lot of questions, who quickly integrated herself into the discounts, into all the transmissions. She would ask us how she could help us, bring us new elements, etc. From there, she quickly made contact with the patients.* (R1).

Initial fears about protective attitudes towards the psychological vulnerability of the peer practitioner were quickly dispelled:

*I did not know what had brought her to consult as a patient and then to make a false step and then to re-invoke a crisis. I was a bit apprehensive at the beginning, that I might be the trigger for a crisis and then finally, that was not the case.* (R4).

### 3.2. Benefits for the patients

The identification process is seen as an unquestionable element for entering a relationship with the patients and establishing a bond of closeness and trust in that “*there is a kind of in-betweenness that is formed*” (R1). Thus, “*it is closer in the end*” (R12) and “*getting into the relationship is perhaps easier with patients*” (R7) who “*feel more listened to*” (R6). The quality of the information obtained was also marked by the specificity of this relationship, as “*she manages to bring out things that we cannot or do not easily address with patients*” (R4).

*This allows us to have someone who is really there to talk, to focus on the person, their feelings, how they really feel, something open, expressive, without necessarily always having to deal with treatments or setting. It is much easier for the patient to open up, to be authentic. We have much more access to the patient’s experience, to how they feel, and as a result, they too can express themselves better.* (R1).

They also reported that the PMHP “*finally has more freedom when she enters into a relationship with a patient*” (R7) because of the absence of medical liability.

*There’s less at stake also in terms of the themes and topics that are discussed and so there will probably be less of that notion of conflict in the relationship that there might be with a caregiver or a doctor or another professional.* (R7).

Regarding her experiential knowledge, they stated that “*in terms of credibility, she has an advantage over us*” (R12). This also rendered her with the legitimacy to confront the patient.

*She has this kind of legitimacy or I think that the patients have a better experience of the fact that she confronts them more.* (R8).

The identification process was seen by caregivers as a powerful way to embody recovery and convey hope to patients.

“*She can say: I’ve been there, you’ll see we’ll get through it. That’s what brings hope, that is to say that there is an identification and then at the same time, she is at the next stage. Or at least, it shows that there is a next step, whereas I realize that when I tell the patient: I know you are going to come out of this, I know it’s a phase; often they look at me and say: how can you know?*” (R9).

Her interventions helped support the other important dimensions of recovery, including knowledge of oneself, one’s resources, and one’s limits.

*Putting words to it, they are more open I have the impression to do the work like that, to put words in fact on what is a resource, what is problematic, what can cause crisis etc. when they are in contact with it.* (R7).

With respect to recovery, her experiential knowledge lends credibility to her interventions.

“*Well, when she, she works with them to set up a whole series of safety nets to avoid hospitalizations; concretely, she has been through this and it makes her speech in this preparation of the discharge project very credible in fact, for them.*” (R12).

In addition to interventions that supported the recovery process, caregivers reported that she played an important advocacy role in enabling patients to better enforce their choices by “*inviting* them to *express their opinions*” (R3) and by helping “to *advocate for the patient’s position in a slightly more thorough way than the caregivers*” (R7).

“*It’s the fact that she can say, she says it often: me, I’ve been a patient, I’ve been in her shoes, so I can understand what she’s going through; so, I do not understand why you are acting like that.*” (R5).

She used different means to perform this role. She acted as “*the advocate, the patient’s representative*” (R12), prepared the interviews with the patients by helping them to “*structure a little what the priorities were*” (R8) or acted as a mediator. The caregivers noted that she “*has a very militant side, of wanting to help as much as possible*” (R7), especially *concerning* “*the drug treatments*” (R12).

Her in-depth knowledge of the network was noted as a benefit for the patients: “*she gives out brochures, she is clear about all the possible follow-ups, and they are quite reassured by this*” (R11). The caregivers mentioned that “*she has other resources that we do not really have that are very important and that we would not have thought of, like support groups for example*” (R4). Her experiential knowledge allowed her to have an accurate view of patient needs at discharge while the caregivers recognized that they were “*hyper-focused on medication, on things, yes, pretty basic*” (R2). Her view was viewed as “*really complementary and valuable*” and that it allowed them to “*broaden their vision*” (R4).

*She has an eye for detail, she really puts herself in their shoes, she imagines the patient in his daily life when he goes out and then there you go. She’ll give him addresses, things, that it’s true that, I would not have thought of.* (R2).

### 3.3. Benefits for the team and institution

The presence of the peer practitioner was regarded as stimulating by the team. She forced the team to step out of their “comfort zone” (R8) by introducing innovative ideas such as “offering CBD replacement therapies for patients who use cannabis” (R9). “With her activist side, she always makes us think, ask questions” (R9). She embodied recovery, which seemed to bring hope to the hospital team:

*It also reminds me that there are also an awful lot of people who are outside of the hospital who are functioning, more or less well but functioning without needing to be hospitalized.* (R3).

Caregivers described the collaboration as positive and constructive, and reported that “the peer practitioner offers a different kind of listening to patients” (R8) and that they did not emphasize the divide but the collaboration between the different professional bodies. They highlighted the benefits in creating a strong bond with patients because she “shares the patients’ experience” (R8) and that patients “feel less judged” (R8). Although she had a different role, she was considered an integral part of the team:

*I find it’s like completing a puzzle. She gives elements, we bring information and then we can understand the situation better. There is a piece missing, she gives it. We can think together.* (R6).

Her presence was also seen as a contribution to changing the image of the hospital and vision of patients in society:

*It gives another image of the institution you are in. Here I am in a hospital that now really takes care of the whole person.* (R1).

The arrival of a peer practitioner helped improve patient engagement and participation in care by providing leverage in the therapeutic relationship.

*I’ve seen with very difficult patients, all of a sudden it’s a kind of trigger, it lifts resistance, it optimizes and then all of a sudden we manage to do something very nice together. It’s really, that is to say that all of a sudden she brings something and we, it’s a lever on us.* (R9).

This bridging role is exemplified by this statement: “she could both represent the health care team to the patient and represent the patient to us” (R12). The team added that the presence of a peer practitioner allowed for a more holistic view of the patient.

*There are certain times when she challenges the way we see things by, putting herself in the patient’s shoes, she rephrases and we think, yeah, maybe we did not see that aspect.* (R2).

### 3.4. Risks

Although the PMHP was successfully integrated and the team reported many benefits for care, her activist stance in defending patient rights could be perceived as a challenge to their practices: “sometimes I have the impression that in certain moments when she is an activist, she cannot put herself in our shoes” (R9). Sometimes her activism led professionals to feel abusive, which was seen as a limitation of the role.

*And, once or twice, there was an team supervision on a patient where it was harder for me because there was a point where her activist side was so powerful that I almost felt like she was saying, but you are abusing her!* (R9).

When the patients presented significant behavioral problems, the peer practitioner found herself at a loss: “all of a sudden she was completely lost, or all her tools were no longer of use because we are in the most extreme setting of psychotic pathology” (R9). Although the collaboration was perceived as positive, the team noted a risk of overlap in the roles of the different professionals: “*there is obviously some overlap in all of this. Sometimes the nurses do the doctors’ work, sometimes the doctor does some of their work. For the PMHP, it’s the same thing*” *(R12)*. This risk seemed to be greater between the nurse and peer practitioner because they worked in a shared environment.

*Sometimes things get a little mixed up and I think that for the team and for her sometimes it’s not very clear what she should or should not do, what we should or should not do. It’s different from the physiotherapist or a doctor who does not work here, who does not work in our office.* (R7).

Several testimonies expressed the fear that the peer practitioners had more time for direct care and enjoyed informal moments because they did not have the administrative and legal burdens experienced by doctors and nurses.

*It is true that it would be a trap to say to ourselves: well, we are putting more peer practitioners and basically the work of the nurses in front of the screens is going to increase even more, or to compensate for the fact that the nurse spends more and more time in front of the screen, we are going to put more peer practitioners.* (R12).

Regarding the professional positioning of the peer practitioner, the team was concerned about the risk of exhaustion linked to emotional over-involvement, especially when confronted with the limits of hospital treatment. Thus, the team might have wanted to protect the peer practitioner, especially during the application of restraining measures: “*we have tended to protect her, so you can see when things are going wrong in the department*” (R9).

*As soon as there is an injustice or what she experiences as an injustice, or the limits of the system simply, yes, sometimes it quickly puts her in all states a little bit or she has trouble accepting if there are limits or if we cannot do everything during a hospital stay.* (R8).

### 3.5. Perspectives

In terms of perspectives, the team considered it important to develop the function in a broader way within the institutional framework: “*I find that it is the future, that it is the work of tomorrow for me and yet I have been in psychiatry for many years*” (R2). Concerning the planning of her activity, they wanted her to be deployed over the whole schedule like the medical and nursing staff; this was meant to favor team cohesion. Indeed, evenings and weekends appeared to provide the best opportunity for relational care outside the usual hospital flow.

*It would almost be beneficial if she worked the same hours as us, if we were really a team, if we had the handover at the same time and if we finished together and if* …*. It’s often when there’s more anxiety; the approach of nightfall triggers*… *That’s perhaps when there are discussions that could be rich.* (R3).

Views were expressed regarding the development of group activities for mutual aid and sharing of experience as well as support for the management of specific symptoms.

*I think everything that is a discussion group, a bit like a forum, so that patients can express themselves and then give us feedback; so that she can be the spokesperson for proposals.* (R3).

The team was invited to question its representations of the function. This aimed to improve their collaboration and enhance the integration process.

*Because indeed, we spent a lot of time understanding what she was doing, which is normal. But what does she perceive? What are her representations also of nurses, doctors? Maybe that’s a difficulty.* (R12).

## 4. Discussion

This study aimed to understand the impact of the integration of the peer practitioner function on a psychiatric inpatient unit as perceived by the multidisciplinary team. The results showed that the introduction of a peer practitioner on a ward was accepted by the team. They felt that the intervention of the peer practitioner helped to convey hope and thus support the recovery process and benefited the patients. These benefits have been reported in other studies to be even more important during hospitalizations, specifically associated with traumatic, coercive, and isolating experiences (3, 4, 6, 19). According to the professionals, the identification process between the patient and the experts may have contributed to this result. This is known to favor a more symmetrical interaction, enabling patients to talk more freely about their problems and to adopt a more optimistic view of themselves (20).

The integration of the peer practitioner function is a source of hope for professionals working in hospital, who recognize that they may be more focused on the crises, illnesses, and drug treatments and are less aware of the potential for recovery. While the very nature of hospital work inevitably leads to a focus on the crisis, it may sometimes lead to their stigmatizing attitude towards patients. Close and frequent contact with individuals who have been affected by mental illness and with those who have recovered has a positive effect on the hospital professionals (21). This supports the relevance of integrating PMHP in the hospital, especially because constant contact with patients in emergency situations may restrict the adoption of an optimistic vision of recovery.

The peer practitioner’s intervention was recognized as having a leverage on the therapeutic relationship between the team and the patient, which helped reduce resistance and improve communication with patients, previously considered as difficult by the team. This leverage effect could be useful to work on relapse prevention with patients through a joint crisis management plan. This shared decision-making process might anticipate the care to be provided in the event of a crisis (22). It must however be mentioned that the construction of a joint crisis plan needs to include outpatient partners considering the prevention of readmission must occur outside of hospital and that sometimes patients are not ready to consider potential readmissions while in hospital. This process can be experienced as anxiety-provoking for patients, could be eased by the integration of a peer practitioner in this collaborative work. However, the effects of PMHP on possible violent behavior or restraint have not been measured in previous studies conducted in hospitals (7–9). Nevertheless, given the effects of the leverage of PMHP on the team-patient relationship, it would be interesting to continue research in this direction; negative interactions between patients and professionals, some of which were defused in the context of the study, are recognized as an important predictor of violent behavior (23).

With regard to the challenges concerning possible areas of tension between the professional fields of activity, the formalization of the role of PMHP should be implemented as recommended in previous study, while remaining vigilant in maintaining a certain flexibility (15, 16). The protective attitudes towards the peer practitioner reflect an ongoing process concerning the status that the team recognizes for them (patient versus colleague). This is a known issue for which it is recommended that the multidisciplinary team be given the same status (16). Certain challenges such as the appropriate level of activism and those concerning the modalities of collaboration within the team, should be addressed in the context of supervision aimed at professional development (14). The respective roles and responsibilities of the nurse and patient could be clarified to enhance a collaborative egalitarian relationship and interdisciplinary coordination among the professionals.

Although the results indicate that nurses recognize the PMHP as a colleague, the egalitarian balance is breached in two ways: first, nurses change their role as colleague to the PMHP when they perceive the PMHP to be vulnerable and feel the need to protect them, such as when they anticipate a risk of burnout caused by the PMHP’s increased involvement or when the PMHP is distraught in complex or extreme care situations, including restraint care. Protective attitudes seem to arise if the nurse is not advised about the triggers of a crisis for the PMHP or is not quickly reassured about the personal management of vulnerabilities by the PMHP. In this case, the nurse may tend to position themselves as caregivers and place the PMHP in a patient role. Second, the collaborative relationship is also disbalanced when the PMHP takes on an advocacy role with the patients. Although advocacy actions are appreciated by the nursing team, they may lead to interprofessional tension if the PMHP speaks out not only “for” the patient (their rights and needs) but also “against” the caregivers and, more broadly, the institution. PMHP should be trained to establish a relationship of trust with the nursing team, for example by explaining the resources for managing emotions, triggers, and signs of personal crisis.

Regarding multidisciplinary professional coordination, the specific areas of responsibility are still unclear. However, it is necessary to first define the specific areas of expertise of the PMHP and to distribute the modalities and actions that will enable them to bring an added value to care. In a spirit of complementarity, the next step would be to define the activities with or without delegation of responsibility. For example, the PMHP is currently recognized to represent an experience of the disease. One of the activities identified as providing added value in care when conducted by a PMHP is the therapeutic discussion group. Could this activity be entrusted to a PMHP and constitute a distinct role? In connection with the protective attitudes of the caregivers mentioned above or an exaggerated involvement of the PMHP, it would be useful to identify the activities or situations in which the PMHP could not be involved in the care, for example during acute or emergency care. Extending this rationale, further analysis could be conducted on the phases or stages of the care trajectory of patients who particularly benefit from the involvement of an PMHP. Moreover, the contributions of a PMHP could be evaluated in terms of patient needs (24).

Nurses and PMHP should also be trained in interprofessional collaboration, with the development of interactional resources that allow for mutual observation and constant adjustment of positions in the professional relationship. Collective team supervision is also a resource for collaborative work. Individual supervision of the PMHP can contribute to integrating the PMHP into the care teams. The PMHPs would benefit from a reflective and formative environment in the field of affectivity management in the workplace, notably by demonstrating an analytical viewpoint at personal involvement (e.g., its observables, variations, triggers). Supervision is a central resource in the development of professionalism in psychiatry (25), particularly by working on emotional involvement and on power and expertise issues in professional relations (with colleagues and patients).

The results of this study are limited by its small sample size, focusing on a single care unit, and relying solely on the perspectives of the professionals involved. The findings may not be representative of other care units with different culture, patient demographics, and staff dynamics. Other stakeholders, such as patients, their families, or external experts, may offer different insights or perspectives that could provide a more comprehensive understanding of the integration of PMHPs. Further study should incorporate multiple perspectives to provide a more comprehensive understanding of the challenges and benefits associated with integrating PMHPs in the hospital environment.

This study was conducted in a hospital unit comprising patients suffering from severe psychiatric disorders associated with behavioral problems. The participants (hospital professionals) reported benefits that reinforced the feasibility of integrating the PMHP function in the hospital unit with patients requiring acute care. The challenges to the integration relate more to the status of the function as well as to collaboration issues, rather than to the benefits for the patients. This shows that professionals are generally open to such developments and that there is a change in basic assumptions regarding the legitimacy of experiential knowledge in proving care. Future studies should focus on the management and supervision of the teams to establish this new status and recognize its full scope of action, as well as on the validation of this new profession at the administrative level where resistance has remained despite the structured training and a large body of scientific literature.

## Data availability statement

Anonymized transcripts supporting the conclusions of this article will be made available by the authors.

## Ethics statement

Ethical approval from the Swiss Human Research Act was not required because no personal data concerning human diseases and the structure and function of the human body were collected. Therefore, this study did not need to be authorized by Swiss ethics. All participants provided their written informed consent. Focus group recordings were transcribed and anonymized verbatims were entered in NVivo 1.7.1.. The studies were conducted in accordance with the local legislation and institutional requirements. The participants provided their written informed consent to participate in this study.

## Author contributions

PL, CS, and JF conceptualized and designed the study and analyzed and interpreted the data for the work. PL and CS acquired the data. PL, CS, JF, and AN drafted the first version of the work. RB, LA, BR, RM, and PC revised it critically for important intellectual content and provided approval to publish the content. PL, CS, RM, RB, LA, BR, PC, AN, and JF agree to be accountable for all aspects of the work in ensuring that questions related to the accuracy or integrity of any part of the work are appropriately investigated and resolved. All authors contributed to the article and approved the submitted version.
